# The orally bioavailable allosteric CXCR4 HIV-1 entry inhibitor AMD11070

**DOI:** 10.1186/1742-4690-9-S1-P7

**Published:** 2012-05-25

**Authors:** Simon Fricker, Renee Mosi, Virginia Anastassova, Jean Labrecque, Rebecca Wong, Renato Skerlj, Gary Bridger, Dana Huskens, Dominique Schols

**Affiliations:** 1 Genzyme Corporation, Framingham, MA, USA; 2 Formally of AnorMED Inc., Langley, BC, Canada; 3 Rega Institute for Medical Research, University of Leuven, Leuven, Belgium

## 

In order to enter and infect human cells HIV must bind to the CD4 receptor in addition to either CXCR4 or CCR5. AMD11070 was the first orally available small molecule inhibitor of CXCR4 to enter the clinic. Here, we report in detail the molecular pharmacology of AMD11070 which is a potent inhibitor of X4 HIV-1 replication in various CD4+ T cell lines, CXCR4-transfected cell lines and in PBMC (IC50 values of 14 ± 3 nM). In addition, AMD11070 potently inhibited cell fusion between a CHO-K1 cell line expressing viral gp120 and the P4-R5 MAGI cells which express CD4 and CXCR4 with an IC50 of 1.5 ± 0.3 nM. No antiviral activity was observed with AMD11070 against CCR5-using (R5) HIV-1 replication. Using CD4+ T cell lines that endogenously express CXCR4 we demonstrate that AMD11070 is an antagonist of CXCL-12 (SDF-1a)-ligand binding (IC50: 12.5 ± 1.3 nM), inhibits CXCL-12-mediated signaling (IC50: 9 ± 2 nM) and that it inhibits CXCL-12-mediated chemotaxis (IC50: 19 ± 4 nM). AMD11070 does not inhibit chemokine-induced Ca2+-signaling in cells expressing CXCR3, CCR1, CCR2b, CCR4, CCR5 or CCR7, demonstrating the compound selectivity for the CXCR4 receptor. In addition, AMD11070 is able to inhibit the SDF-1beta isoform interactions with CXCR4 and N-terminal truncated variants of CXCR4 with equal potency as to the wild type CXCR4 receptor. These data indicate that AMD11070 is an allosteric antagonist of CXCR4. A proof-of-concept clinical trial has shown that AMD11070 can reduce the viral load of X4 HIV-1 in HIV-1-infected persons. Together these data further support to the potential beneficial role of orally bioavailable CXCR4 inhibitors as a therapeutic option for HIV/AIDS treatment.

